# Atopic dermatitis in amyotrophic lateral sclerosis successfully treated by dupilumab: A case report

**DOI:** 10.1097/MD.0000000000043737

**Published:** 2025-08-01

**Authors:** Qian Zhang, Xiyuan Zhou, Ge Yang, Lixia Zhang

**Affiliations:** a Dermatology, The Fourth Affiliated Hospital of Southwest Medical University (Tianfu Affiliated Hospital), Meishan, China; b Institute of Dermatology and Venereology, Sichuan Provincial People’s Hospital, University of Electronic Science and Technology, Chengdu, China.

**Keywords:** amyotrophic lateral sclerosis, atopic dermatitis, dupilumab

## Abstract

**Rationale:**

Atopic dermatitis (AD) is a common skin disorder characterized by symmetric erythematous papules in flexural areas, with pruritus of varying intensity persisting throughout its course. Amyotrophic lateral sclerosis (ALS) is a neurological disease with progressive loss of muscle function, and its treatment remains a challenge worldwide. When patients suffer from both conditions, the skin issues are often overlooked by both physicians and family members. However, the pruritus becomes unbearable for the patients, particularly as they experience a progressive loss of the ability to scratch autonomously.

**Patient concerns:**

An elderly 75-year-old male patient with comorbid ALS and AD, suffering from prolonged pruritus, had lost faith in all topical and oral medications.

**Diagnosis:**

AD and ALS.

**Interventions:**

The patient received subcutaneous dupilumab with an initial 600 mg dose followed by 300 mg every 2 weeks, and was monitored for 12 weeks during follow-up.

**Outcomes:**

The patient exhibited gradual reduction in pruritus severity and skin lesions throughout the treatment course. No adverse reactions other than mild conjunctivitis were reported.

**Lessons:**

This case demonstrates the successful application of dupilumab in an AD patient with comorbid ALS. It not only provides a clinically instructive case reference for dupilumab therapy in AD, but also underscores that pruritus in ALS patients warrants greater clinical attention.

## 1. Introduction

Atopic dermatitis (AD) is a chronic inflammatory skin disease in which itching is the main symptom. Scratching is difficult to avoid throughout the course of the disease. Amyotrophic lateral sclerosis (ALS) is a fatal neurodegenerative disease characterized by progressive deficits in muscle function, owing to pathological protein deposition in the cytoplasm of motor neurons.^[1]^ Patients with ALS experience symptoms of muscle weakness however, touch, pain, and itching sensations remain normal.^[2]^

## 2. Case report

A 75-year-old male patient had recurrent erythematous macules and papules with itching mainly on the flexed side of the body for more than 2 years. Combined history of asthma and allergic rhinitis for more than 20 years. Thus diagnosed with moderate AD according to the Hanifin and Rajka and Scoring Atopic Dermatitis scales.^[3]^ ALS was diagnosed according to the revised El Escorial criteria^[4]^ 3 years prior to presentation; he was currently on riluzole (50 mg), administered twice daily. The pharyngeal reflex was weakened, and the muscle strength of the extremities was 0. The body was covered with dry skin, with a few visible fine white scales; red spots of varying sizes were scattered on the back, and millet grain to mung bean-sized papules were distributed. The erythema on the instep is eroded with yellow exudate and crusts. Laboratory tests revealed that the blood C-reactive protein was 81.92 mg/L (normal: 0–5 mg/L), immunoglobulin E was 258 IU/mL (normal: 0–100 IU/mL), and erythrocyte sedimentation rate was 65 mm/h (normal: 0–15 mm/h). Routine blood tests revealed no abnormalities in the liver and kidney functions, and blood lipids. Dupilumab injections (600 mg at week 0, followed by 300 mg every 2 weeks) were administered to treat the patient’s AD. After 12 consecutive weeks of treatment, the patient’s pruritus was significantly relieved and the lesions largely subsided (Table 1). Only at about week 8 did the patient develop a small number of new erythematous spots and papules on the face and scalp after exposure to the pollen, and the itching worsened slightly. These symptoms were quickly relieved after leaving the sensitized environment and adding oral (10 mg daily) and topical “dexamethasone boric acid cream.” The whole process of dupilumab treatment was accompanied by the original ALS regimen, and no other adverse reactions and related effects on ALS were observed except for mild conjunctivitis.

**Table 1:** Patients’ scores before and every 2 weeks after treatment with dupilumab.

Time (week)	SCORAD	ADCT	NRS
0	28.8	21	6
2	16.2	15	4
4	11	7	3
6	9.4	4	2
8	12.4	7	3
10	11	5	2
12	8.6	4	2

ADCT = Atopic Dermatitis Control Tool, NRS = Pruritus Numerical Rating Scale, SCORAD = Scoring Atopic Dermatitis.

## 3. Discussion

ALS’s exact pathological mechanism remains unclear, and existing treatments can only slow disease progression to a certain extent, with patients predictably experiencing a gradual loss of muscle function throughout the body until death.^[2]^ Patients with ALS have symptoms related to skeletal muscle weakness, while touch, pain, and itching sensations remain normal. Sympathetic nerve damage may be accompanied by skin numbness, itching, and abnormal sweating, among others.^[5]^ Therefore, they have no choice but to tolerate the itching caused by AD. Psycho-emotional problems such as depression, anxiety, and sleep disorders are more prominent in these patients; therefore, it is necessary to alleviate AD-related symptoms to improve their quality of life. Various emerging biological agents have shown obvious advantages over other methods for skin disease treatment. Among them is dupilumab, the first drug approved by the FDA for systemic AD treatment in 2017, opening up a new era of biological AD treatments.^[6]^ Dupilumab is a fully human monoclonal antibody against IL-4Rα that blocks IL-4 and IL-13 signaling and has been shown to have good long-term efficacy and safety in treating children, adults, and older adult patients with AD in numerous clinical trials worldwide.^[7]^ According to the diagnostic criteria of Hanifin and Rajka and the Scoring Atopic Dermatitis scale,^[3]^ the patient was diagnosed with moderate AD. Because ALS had progressed to a state of autonomic movement, the patient suffered from unbearable itching and lost confidence in traditional treatment; therefore, we administered dupilumab, achieving the expected clinical results.

In 2022, Kiyohara et al^[8]^ reported the case of a female patient with a history of AD for over 20 years who developed ALS at the age of 51 years. As the patient’s muscular dysfunction worsened, the AD symptoms significantly reduced, and the recalcitrant lesions on the arms gradually disappeared. The difference between our case and the case above is that the AD-related manifestations and symptoms appeared after the initial ALS diagnosis. According to the patient and the family’s descriptions, AD appears to have gradually worsened along with the progression of the ALS. It is unclear how ALS-related muscle hypoplasia or deficiency affects AD pathophysiology, signs, and symptoms in patients with AD together with ALS.

## 4. Conclusion

Our study confirms that dupilumab treatment in patients with AD together with ALS is effective and safe and does not appear to impact ALS progression and treatment. It should be noted, however, that this is a single-case report with limited follow-up duration. Furthermore, posttreatment laboratory investigations were not obtained due to the patient’s travel barriers and socioeconomic constraints, thereby compromising the comprehensive analysis of clinical data. Thus, further investigation into dupilumab application for such rare comorbid cases will undoubtedly require larger clinical cohorts and more comprehensive follow-up monitoring.

## Author contributions

**Conceptualization:** Qian Zhang, Xiyuan Zhou, Ge Yang, Lixia Zhang.

**Data curation:** Qian Zhang, Xiyuan Zhou, Ge Yang, Lixia Zhang.

**Formal analysis:** Qian Zhang, Lixia Zhang.

**Funding acquisition:** Qian Zhang, Lixia Zhang.

**Investigation:** Qian Zhang, Xiyuan Zhou.

**Methodology:** Qian Zhang, Xiyuan Zhou.

**Project administration:** Qian Zhang, Xiyuan Zhou, Ge Yang.

**Resources:** Qian Zhang, Lixia Zhang.

**Software:** Qian Zhang, Ge Yang.

**Supervision:** Qian Zhang, Xiyuan Zhou, Ge Yang.

**Validation:** Qian Zhang, Lixia Zhang.

**Visualization:** Qian Zhang, Ge Yang, Lixia Zhang.

**Writing – original draft:** Qian Zhang.

**Writing – review & editing:** Qian Zhang, Xiyuan Zhou, Lixia Zhang.
